# Molecular Design of Catechol-Containing Phospholipid Polymers toward Effective Functionalization of Magnetic Nanoparticles for Cancer Hyperthermia

**DOI:** 10.1021/acsomega.5c02791

**Published:** 2025-07-11

**Authors:** Masahiro Kaneko, Kanato Yukishita, Kaname Tsutsumiuchi, Akira Ito

**Affiliations:** † Department of Chemical Systems Engineering, Graduate School of Engineering, 12965 Nagoya University, Furo-cho, Chikusa-ku, Nagoya 464-8603, Japan; ‡ College of Bioscience and Biotechnology, 12740 Chubu University, 1200 Matsumoto, Kasugai, Aichi 487-8501, Japan

## Abstract

Cancer hyperthermia induced through magnetic nanoparticles that generate heat upon irradiation with an alternating magnetic field (AMF) allows local heating of tumor tissues, leading to cancer cell death. For the clinical application of magnetic nanoparticles, designing an appropriate surface structure is important for maintaining colloidal stability and stealth properties that prevent clearance by the mononuclear phagocyte system. Catechol-containing polymers offer stable modification and functionalization of nanoparticles. However, the effect of their molecular structure on the modification efficiency and function of nanoparticles remains unclear. Herein, magnetite nanoparticles (MNPs) modified with a series of catechol-containing polymers composed of dopamine methacrylamide (DMA) units and biocompatible phosphorylcholine units were prepared. Higher molecular weights of the polymers resulted in higher modification amounts on the MNPs, whereas the DMA unit content had little impact. Modifying the polymers improved the dispersion stability of the MNPs in phosphate-buffered saline and their stealth property against macrophages in vitro. Polymers with more than 1.8 mol % DMA units enabled stable dispersion of MNPs for 14 days. Modifying the polymers with DMA unit contents between 4.8 and 9.0 mol % minimized the macrophage uptake of the MNPs. Moreover, the polymer-modified MNPs were loaded with the anticancer drug bortezomib, and the release of bortezomib was enhanced by irradiation with an AMF for magnetic hyperthermia. In vitro magnetic hyperthermia with polymer-modified MNPs successfully killed mouse colon cancer cells, and bortezomib loading augmented the anticancer activity. This study will provide crucial guidance on the molecular design of catechol-containing polymers for effective cancer hyperthermia using magnetic nanoparticles.

## Graphical abstract

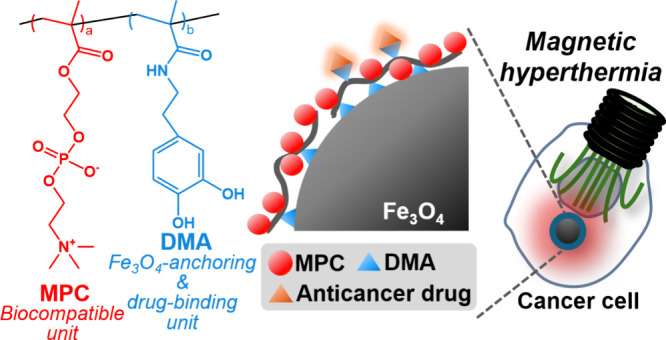

## Introduction

Cancer hyperthermia refers to heating cancer cells above 42.5 °C, leading to cell death. Magnetic nanoparticles capable of generating heat under an alternating magnetic field (AMF) exhibit potential for selective heating of tumor tissue.^1–4^ Intravenously administered nanoparticles ranging from 20 to 200 nm tend to accumulate in tumor tissue due to the enhanced permeation and retention (EPR) effect.^5,6^ However, exogenous particles suffer from aggregation in blood and are entrapped and excreted by the mononuclear phagocyte system as a foreign body reaction, resulting in insufficient accumulation in tumor tissues.^7^ Therefore, the molecular design of the surface of magnetic nanoparticles to enhance colloidal stability and impart stealth characteristics is critical for prolonging blood circulation and maximizing accumulation in tumors via the EPR effect, thereby enabling effective magnetic hyperthermia.

Modifying magnetic nanoparticles with synthetic polymers shows promise for improving their colloidal stability, biocompatibility, and therapeutic efficacy. The selection of particle-binding groups is vital for stable polymer modification in nanoparticles. Magnetic nanoparticles are generally composed of transition metals or metal oxides.^8^ In particular, magnetite (Fe_3_O_4_) nanoparticles (MNPs) have gained attention owing to their chemical stability and minimal toxicity and have been clinically applied as a contrast agent for magnetic resonance imaging.^9–11^ Carboxy, phosphate, and catechol groups have been utilized for the surface modification of MNPs.^11–14^ In particular, catechol-containing polymers, inspired by mussel adhesive proteins, are stably immobilized on iron oxide surfaces.^15,16^ The effect of the structure of catechol-containing polymers on adhesion to planar substrates has been actively studied for polymer glues.^17–20^ Synthetic polymers with multiple catechol units in a single polymer chain presumably exhibit more stable anchoring to nanoparticles than those with only one catechol group in a single polymer chain, owing to their multidentate property.^21,22^ Therefore, catechol-containing polymers with multidentate bonding capacity can effectively modify and functionalize MNPs. However, the effect of the structure of catechol-containing polymers on the modification efficiency and properties of the nanoparticles remains unclear.

The combination of hyperthermia and chemotherapy is a promising approach for providing potent anticancer activity.^23^ Typically, chemotherapy exhibits poor therapeutic efficacy and side effects owing to its toxicity to normal cells.^24,25^ The loading of drugs onto nanoparticles allows the accumulation of drugs in tumors based on the EPR effect and enables the combination of hyperthermia and chemotherapy. Catechol groups can reversibly bind to drugs containing boronic acid; this binding can be dissociated by heating for drug release.^26,27^ Because MNPs can be heated by AMF irradiation from outside the patient’s body, drug-bound MNPs with catechol groups can release drugs triggered by AMF irradiation. Thus, catechol units can be utilized not only for stable binding to MNPs but also for drug release via AMF irradiation.

Among stealthy synthetic polymers, polyethylene glycol (PEG) has been widely used to modify the surface of magnetic nanoparticles for biomedical applications.^28–30^ However, due to widespread exposure to PEG through pharmaceuticals, cosmetics, and food additives, anti-PEG antibodies are increasingly observed in the general population.^31–33^ This response raises concerns that PEG-based materials may experience reduced efficacy or accelerated blood clearance when administered systemically. In this context, zwitterionic polymers, particularly those containing phosphorylcholine moieties, have gained attention as promising alternatives due to their excellent biocompatibility and potentially lower immunogenicity.^34,35^ In this study, we synthesized a series of random copolymers of poly­[2-methacryloyloxyethyl phosphorylcholine (MPC)-*co*-dopamine methacrylamide (DMA)] (PMD). PMD comprises catechol-containing DMA and zwitterionic MPC units. MPC is a biocompatible molecule inspired by the polar groups of phospholipids, particularly those containing phosphorylcholine moieties, and the modification of artificial materials with MPC polymers suppresses foreign body reactions.^36,37^ We prepared MNPs modified with PMD (MNPs@PMD) and investigated the effect of the catechol unit content and molecular weight of PMD on the amount of modification of MNPs, dispersion stability of nanoparticles, and stealth effect on macrophages. In addition, we loaded the anticancer drug bortezomib (BTZ) onto MNPs@PMD to examine its release characteristics and the in vitro therapeutic effects of magnetic hyperthermia.

## Materials and Methods

### Chemicals

MPC, DMA, and 2,2’-azodiisobutyronitrile (AIBN) were purchased from Sigma-Aldrich (USA), Specific Polymers (France), and Fujifilm Wako Pure Chemical (Japan), respectively. All organic and inorganic reagents were commercially available and of extra pure grade and were used without further purification.

### Synthesis of MNPs

The MNPs were synthesized using a coprecipitation method. An aqueous iron ion solution (300 mL) consisting of 39.8 g of FeCl_2_·4H_2_O and 108 g of FeCl_3_·6H_2_O was added to 700 mL of a 4.0 mol/L NaOH solution by stirring using a homogenizer (Ultra-Turrax T25 IKA, Germany) at 12,000 rpm. The solution was stirred for 1 min, centrifuged at 6000 rpm for 1 min, and washed with pure water. Centrifugation was repeated until the supernatant became turbid. The sediment was dispersed in 150 mL of pure water. Fifteen zirconia balls (diameter: 20 mm) and 300 g of zirconia balls (diameter: 1 mm) were added to the suspension, sonicated with a Branson Sonifier 450D (Emerson, USA) for 30 min, and shaken for 5 days. The resulting black suspension was centrifuged at 10,000 rpm for 10 min, and the supernatant was removed and resuspended in pure water. The MNPs were observed by transmission electron microscopy (TEM; JEM-2100Plus, JEOL, Japan) and analyzed by X-ray diffraction (XRD; Aeris, Malvern Panalytical, U.K.). The particle size distribution was determined by using ImageJ software to calculate the equivalent circular diameters of nanoparticles with identifiable outlines, selected from three TEM images. The magnetic property of MNPs was evaluated using a vibrating sample magnetometer (VSM) option (P525, Quantum Design, USA) of a physical properties measurement system (PPMS-9, Quantum Design) at 300 K.

### Synthesis of PMD

PMD and poly­(MPC) (PMPC) were synthesized by free-radical polymerization using AIBN as an initiator. PMD1, PMD2.5, PMD5, PMD10, PMD30, and PMPC were synthesized by varying the feed ratio of MPC to DMA, as shown in Table 1. PMD1-L, PMD30-H, PMPC-H1, and PMPC-H2 were synthesized by varying the MPC and AIBN concentrations. As a representative, PMD30 was synthesized as follows: In a test tube, 2.07 g (7.00 mmol) of MPC, 0.664 g (3.00 mmol) of DMA, and 0.0164 g (0.100 mmol) of AIBN were added with 20 mL of a mixture of ethanol/water = 1/1 (v/v). The solution was purged with argon gas for 15 min and polymerized at 60 °C for 24 h. After the polymerization, the solution was reprecipitated using acetone. The precipitate was dissolved in pure water and dialyzed for 3 days using a Spectra/Por 7 membrane (MWCO 3.5k, Spectrum Laboratories, USA). Subsequently, the polymer solution was lyophilized, and the resultant powder was obtained as PMD30. The compositions of the MPC and DMA units were calculated by ^1^H NMR measurement using a mixture of D_2_O/C_2_D_5_OD = 1/1 (v/v) (Figure S1). The molecular weight of PMD was evaluated by gel permeation chromatography (GPC) using an OHpak SB-805 HQ column (Resonac, Japan) and a refractive index detector (RI-4030, JASCO, Tokyo, Japan) using a 0.5 M sodium acetate/0.5 M acetic acid buffer^38^ as an eluent and PEG standards (*M*_p_ = 0.64k–1039k, Agilent Technologies, USA) as standards. The molecular weight of PMPC was evaluated by GPC with an OHpak SB-805 HQ column and a refractive index detector using a mixture of water/methanol = 7/3 (v/v) containing 10 mM LiBr as the eluent and PEG standards (*M*_p_ = 0.64k–1511k) as the standard.

**Table 1:** Properties of the Polymers Synthesized in This Study

	in feed (mol %)MPC/DMA^a^	monomer concentration (M)	AIBN concentration (mM)^b^	polymerization (h)	in copolymer (mol %)MPC/DMA^c^	*M*_w_ (×10^4^)^d^	*M*_w_/*M*_n_^d^
PMPC	100/0	0.5	0.5	6	100/0	7.2	2.5
PMPC-H1	100/0	0.5	0.05	6	100/0	13.9	1.9
PMPC-H2	100/0	2	0.025	0.25	100/0	115.3	2.7
PMD1	99/1	0.5	0.5	24	99.4/0.6	66.6	6.4
PMD1-L	99/1	0.5	5	24	99.1/0.9	18.6	4.4
PMD2.5	97.5/2.5	0.5	0.5	24	98.2/1.8	56.9	5.8
PMD5	95/5	0.5	0.5	24	95.2/4.8	51.9	6.6
PMD10	90/10	0.5	0.5	24	91.0/9.0	38.1	6.0
PMD30	70/30	0.5	0.5	24	74.1/25.9	10.2	2.6
PMD30-H	70/30	0.5	0.05	24	76.2/23.8	96.2	6.4

a MPC: 2-methacryloyloxyethyl phosphorylcholine; DMA: dopamine methacrylamide.

b AIBN: 2,2’-azodiisobutyronitrile.

c Determination by ^1^H NMR spectroscopy.

d The number-average (*M*_n_) and weight-average (*M*_w_) molecular weights were measured by gel permeation chromatography (GPC) using polyethylene glycol standards. The eluents were a mixture of water/methanol = 7/3 (v/v) containing 10 mM LiBr for PMPC and 0.5 M sodium acetate/0.5 M acetic acid buffer for PMD.

### Preparation of Polymer-Modified MNPs

MNPs were modified with PMD and PMPC to prepare MNPs@PMD and MNPs@PMPC, respectively. MNPs@PMD30 was prepared as follows: Three milliliters of the MNP dispersion (5.0 mg*Fe_3_O_4_*/mL) in pure water were mixed with 3 mL of a PMD30 aqueous solution (167 mg/mL). The suspension was stirred for 1 h and centrifuged at 10,000 rpm for 20 min. The precipitate was washed with pure water, centrifuged at 10,000 rpm for 1 h, and resuspended in pure water. When necessary, the dispersions were lyophilized to obtain dried samples. The hydrodynamic size of the MNPs was evaluated by analyzing an aqueous suspension of MNPs (0.10 mg*Fe_3_O_4_*/mL) using dynamic light scattering (DLS; Zetasizer Nano ZS, Malvern Panalytical). The amount of modified polymers was evaluated using thermogravimetric analysis (TGA) with DTG-60AH (Shimadzu, Japan) under a nitrogen atmosphere while the temperature was increased to 900 °C at increments of 10 °C/min, and the loss of mass between 250 and 600 °C was calculated.

### Determination of Concentration of Magnetite

The concentration of MNPs was evaluated by quantifying the iron ions. To dissolve the MNPs, 400 μL of the MNP dispersion was mixed with 200 μL of 12 N HCl and incubated in a water bath at 40 °C for 30 min. Thereafter, 10 μL of hydrogen peroxide and 4 mL of 0.1 M potassium thiocyanate were added to the solution and vortexed. The absorbance was measured at 480 nm using a spectrophotometer. The iron concentration was determined using an iron standard solution (Nacalai Tesque, Japan).

### Evaluation of Colloidal Stability of MNPs

Polymer-modified MNPs were dispersed in phosphate-buffered saline (PBS) at a concentration of 2.0 mg*Fe_3_O_4_*/mL. Photographs of glass vials containing 1 mL of the dispersions were captured after 1, 4, 7, 14, 25, and 29 days.

### Measurement of Heat Generation of MNP Dispersions under AMF Irradiation

Glass vials containing 1 mL of the MNP dispersion in pure water (2.0 mg/mL) were irradiated with an AMF for 5 min at a frequency (*f*) of 350 kHz, and a magnetic field amplitude (*H*) of 9.5 kA/m. The product of frequency and magnetic field amplitude (*fH*) was 3.3 × 10^9^ A/m·s, which is below the commonly cited biological safety limit of 5 × 10^9^ A/m·s.^39,40^ An optical fiber thermometer (Anritsu Meter, Japan) was inserted into the vial to monitor the temperature elevation (Δ*T*). The temperature elevation versus time (Δ*T*–*t*) curve was fitted with the Box-Lucas model:

$$\Delta T=T_{\max}\left(1-e^{-\lambda t}\right)$$

where *T*_max_ is the maximum temperature elevation, and λ is the heat transfer rate from the sample to the surroundings.^41^ Model parameters were determined using the least-squares method with the Solver function in Excel (Microsoft, USA).

From the fitted curve, the initial rate of temperature increase, i.e., the first derivative at *t* = 0 was calculated as follows:

$${\left(\frac{d\Delta T}{dt}\right)}_{t\to 0}=T_{\max}\cdot \lambda$$

The specific absorption rate (SAR) of bare MNPs was determined using the following equation:^41,42^

$$\mathrm{SAR}[W/gFe_{3}O_{4}]=\frac{C_{w}\cdot m_{w}}{m_{Fe3O4}}\cdot {\left(\frac{d\Delta T}{dt}\right)}_{t\to 0}$$

where *m*_w_ and *m*_*Fe3O4*_ represent the mass of water and MNPs, respectively, and *C*_w_ is the specific heat capacity of water (4.18 J/gK).

Based on the SAR value, the intrinsic loss power (ILP) of the bare MNPs was calculated using the following equation:^43^

$$\mathrm{ILP}[nH\cdot m^{2}/{\mathrm{kg}}_{Fe3O4}]=\frac{\mathrm{SAR}}{H^{2}\cdot f}$$

### Cell Culture

The mouse colorectal carcinoma cell line CT26 (CRL-2638) was purchased from the American Type Culture Collection (ATCC). CT26 cells (1.0 × 10^6^ cells) were seeded in a 100 mm tissue culture dish in 10 mL of RPMI1640 (Fujifilm Wako Pure Chemical) supplemented with 1% penicillin–streptomycin (Fujifilm Wako Pure Chemical) and 10% fetal bovine serum (FBS) (Funakoshi, Japan) and incubated in a CO_2_ incubator (5% CO_2_ in air) at 37 °C. CT26 cells were passaged every 2 or 3 days.

The mouse leukemic monocyte macrophage cell line RAW264 (RCB0535) was purchased from the RIKEN BRC through the National Bio-Resource Project of the MEXT/AMED, Japan. RAW264 cells (1.0 × 10^6^ cells) were seeded in a 100 mm polystyrene dish in 10 mL of E-MEM (Fujifilm Wako Pure Chemical) with 1% penicillin–streptomycin, 10% FBS, and 1% l-glutamine solution (200 mM, Sigma-Aldrich). RAW264 cells were incubated in a CO_2_ incubator (5% CO_2_ in air) at 37 °C and were passaged every 2 or 3 days.

### Evaluation of Uptake of MNPs into RAW264 Cells

In a 60 mm tissue culture dish, 3 mL of RAW264 cell suspension (5.0 × 10^5^ cells/mL) in E-MEM was added and incubated for 24 h. After incubation, the medium was replaced with 3 mL of E-MEM containing bare MNPs or polymer-modified MNPs (0.10 mg*Fe*_3_ *O*_4_/mL) and incubated again for 24 h. Thereafter, the medium was removed, the cells were washed twice with 2 mL of Dulbecco’s phosphate buffered saline (DPBS; Fujifilm Wako Pure Chemical) to remove the noninternalized nanoparticles. Next, 2 mL of trypsin was added and incubated for 5 min. EMEM was added, and the cell suspension was collected. The cell suspension was centrifuged at 1000 rpm for 5 min, and the supernatant was removed. HCl (12N, 200 μL) was added to the cell pellet and incubated in a refrigerator (4 °C) for 24 h. One milliliter of 5% trichloroacetic acid was added, and the suspension was placed in a refrigerator for 30 min. The suspension was centrifuged at 10,000 rpm for 20 min, and 400 μL of the supernatant was harvested. To the supernatant, 10 μL of hydrogen peroxide and 4 mL of 0.1 M potassium thiocyanate were added, and the solution was vortexed. The absorbance at 480 nm was measured using a spectrophotometer. The iron concentration was determined using an iron standard solution.

### Preparation of BTZ-Loaded Polymer-Modified MNPs

BTZ-loaded MNPs@PMD were synthesized by adding BTZ to the MNPs@PMD10. Three milliliters of the MNP dispersion (5.0 mg*Fe*_3_ *O*_4_/mL) in pure water were mixed with 3 mL of the PMD10 aqueous solution (167 mg/mL). The suspension was stirred for 1 h and centrifuged at 10,000 rpm for 20 min. The precipitate was washed with pure water, centrifuged at 10,000 rpm for 1 h, and resuspended in 1 mL of pure water. The suspension was mixed with 1 mL of BTZ solution in dimethylformamide (1.0 mg/mL) and stirred for 24 h. To evaluate the BTZ release, the BTZ solution (1.0 mg/mL) in dimethyl sulfoxide (DMSO) instead of dimethylformamide was mixed. The suspension was centrifuged at 10,000 rpm for 1 h, and the precipitate was suspended in pure water. The resulting suspension was designated as BTZ-loaded MNPs@PMD10-BTZ.

### Evaluation of BTZ Release of MNPs@PMD-BTZ

MNPs@PMD10-BTZ were suspended in 1.25 v/v% DMSO in DPBS at 1.0 mg*Fe*_3_ *O*_4_/mL. Two milliliters of the suspension were transferred to a dialysis membrane (Spectra/Por 7 Membrane, MWCO 50 k, Spectrum Laboratories) and immersed in 50 mL of 1.25 v/v% DMSO in DPBS. The suspension was irradiated with the AMF for 30 min. The frequency and maximum power of the AMF were 350 kHz and 8 kW, respectively. The temperature of the suspension was simultaneously monitored using a fiber-optic thermometer (Anritsu Meter) and maintained at 43 °C by controlling the output of the AMF generator. One milliliter of the solution was collected 1, 3, 6, and 24 h after AMF irradiation, and the absorbance of BTZ at 270 nm^26,27^ was measured using a spectrophotometer.

### In Vitro Hyperthermia for CT26 Cells

CT26 cells (5.0 × 10^5^ cells) in 3 mL RPMI1640 were seeded in a 60 mm tissue culture dish and incubated for 24 h in 5% CO_2_ at 37 °C. The RPMI medium was removed, and the cells were washed twice with 1 mL of DPBS. Dispersions of MNPs@PMD10 or MNPs@PMD10-BTZ in DBPS (0.50 mg*Fe*_3_ *O*_4_/mL, 1.0 mL) were added to the CT26 cells. The cells were irradiated with AMF for 30 min at a frequency of 350 kHz and a maximum power of 8 kW. During the AMF irradiation, the temperature of the supernatant was monitored using a fiber-optic thermometer (Anritsu Meter), and the output of the AMF generator was adjusted to maintain the temperature at 43 °C. Subsequently, 3 mL of the RPMI medium was added to the dish and incubated at 37 °C for 24 h. After incubation, the medium was removed and the cells were washed twice with 1 mL of DPBS. The cells were treated with 1 mL of trypsin for 5 min, and 2 mL of RPMI1640 was added. The cell suspension was collected, and cell viability was determined using the trypan blue exclusion method.

### Statistical Analysis

Comparisons between multiple groups were performed using Tukey’s test with EZR (Saitama Medical Center, Jichi Medical University, Japan).^44^ A *p*-value less than 0.05 was considered significant.

## Results and Discussion

### Synthesis and Characterization of Polymer-Modified MNPs

To investigate the effect of the polymer structure on the efficiency of MNP modification, PMD and PMPC with different unit compositions and molecular weights were synthesized via free radical polymerization (Table 1). PMD1-L, PMD30-H, PMPC-H1, and PMPC-H2 were synthesized by varying the concentration of AIBN. Monomers containing catechol groups typically necessitate the use of protective groups during radical polymerization; however, it has been reported that DMA can be polymerized by radical polymerization^45^ and copolymerized with MPC,^38,46,47^ even in the absence of protective groups. Employing monomers without protective groups can simplify the synthetic procedure. Despite the relatively high polydispersity index (*M*_w_/*M*_n_) of the PMDs, each PMD exhibited a unimodal peak in GPC analysis (Figure S2). The molecular weight tended to decrease as the content of the DMA units in the PMD (MPC/DMA) increased. PMD1-L, polymerized with an increased amount of AIBN, had a smaller molecular weight than PMD1, whereas PMD30-H, polymerized with a reduced amount of AIBN, had a larger molecular weight than PMD30. The molecular weights of PMPC increased in the following order: PMPC < PMPC-H1 < PMPC-H2.

MNPs were synthesized via a coprecipitation method with FeCl_3_ and FeCl_2_ as iron­(II/III) precursors and NaOH as a precipitant. TEM analysis revealed that the synthesized particles displayed slightly irregular polyhedral or quasi-spherical morphologies with an average diameter of 12.1 nm (Figure S3). XRD analysis confirmed that the nanoparticles consisted of magnetite (Figure S4). The MNPs exhibited the reversible magnetization curve and nearly zero coercivity, indicating their superparamagnetic behavior, with a saturation magnetization of 63 emu/g (Figure S5). Superparamagnetic iron oxide nanoparticles, including MNPs, generate heat under an AMF through Néel and Brownian relaxation mechanisms.^48^ The SAR and ILP of the MNPs upon AMF irradiation were 220 W/g*Fe*_3_ *O*_4_ and 6.88 nHm^2^/kg*Fe*_3_ *O*_4_ (Figure S6), demonstrating that they were effective for magnetic hyperthermia.^42^

MNPs@PMD was prepared by mixing the MNPs aqueous dispersion with PMD (Scheme 1). The heat generation properties of PMD@MNPs upon AMF irradiation are shown in Figure S6, indicating that the PMD modification did not deteriorate the performance of the MNPs as a nanoheater. The amount of modified polymer on the MNPs was evaluated using TGA (Figure 1). No clear correlation was observed between the DMA unit content and amount of modified polymers (Figure 1a, correlation coefficient = −0.14). In contrast, a positive correlation was observed between the molecular weight of the PMD and the amount of modified polymer (Figure 1b, correlation coefficient = 0.85), indicating the importance of molecular weight for efficient polymer modification. Comparing PMD and PMPC, PMD exhibited a more significant increase in the modification amount as the molecular weight increased (Figure 1b). This result indicated that the presence of DMA units helped increase the modification amount. Collectively, polymers containing DMA units with higher molecular weights resulted in higher modification amounts.

**Scheme 1:**
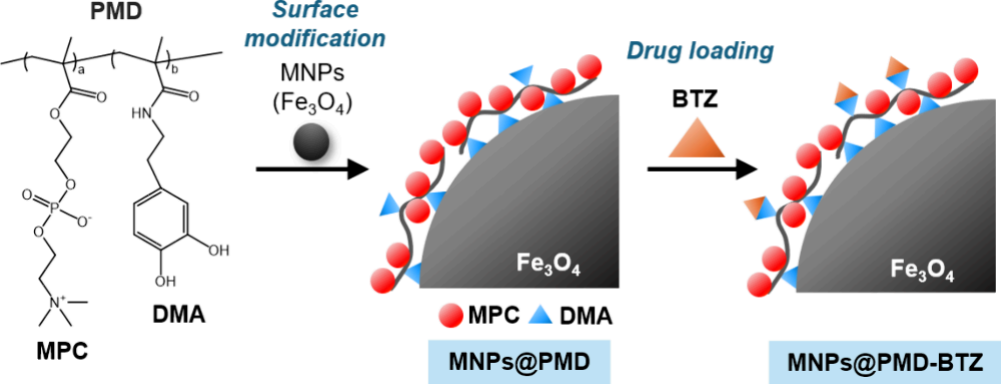
Chemical Structure of Poly­(MPC-*co*-DMA) (PMD) and Schematic of the Preparation of MNPs@PMD and MNPs@PMD-BTZ

**Figure 1:**
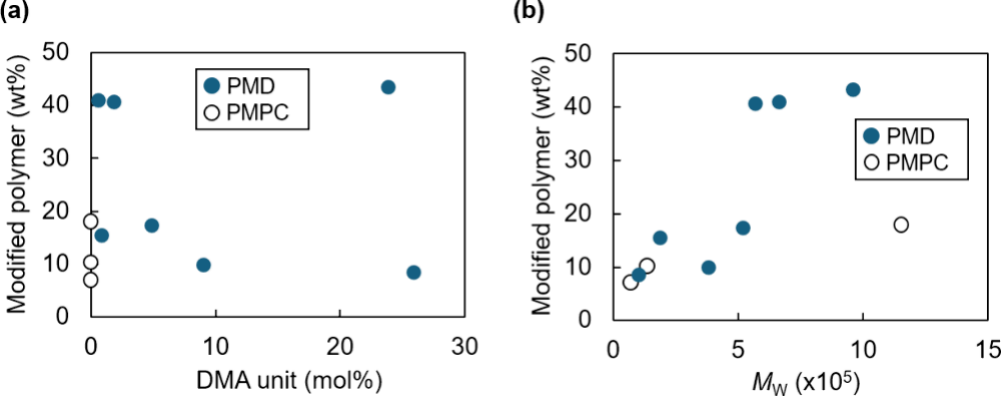
Relationship between the molecular structure and amount of modified polymer on MNPs. (a) Amount of modified polymer on MNPs versus DMA unit content in the polymers and (b) amount of modified polymer on MNPs versus weight-average molecular weight (*M*_W_) of the polymers.

### Colloidal Stability of MNPs

Colloidal stability under physiological conditions is essential for the biological applications of MNPs. The effect of polymer modification of MNPs on the dispersion stability in PBS was evaluated. The bare MNPs precipitated within 1 h, while the polymer-modified MNPs remained dispersed (Figure 2), consistent with previous reports on the improvement of water dispersibility of MPC polymer-modified MNPs.^14,49,50^ Steric stabilization of MNPs by MPC polymer modification,^51^ the unique hydration properties of MPC polymers,^52,53^ and the absence of specific interactions between MPC polymers^54^ contribute to preventing particle agglomeration and improving the colloidal stability of MNPs. MNPs@PMPC and MNPs@PMD1 precipitated on day 4, whereas MNPs@PMD2.5, MNPs@PMD5, MNPs@PMD10, and MNPs@PMD30 remained dispersed, even on day 14. On day 29, all MNPs began to precipitate, whereas MNPs@PMD2.5 was more distinctively precipitated than MNPs@PMD5, MNPs@PMD 10, and MNPs@PMD30. Despite the relatively high modification level of MNPs@PMD1 (41 wt %), its dispersion stability was inferior to that of the other MNPs@PMDs. This result indicates that the modification amount does not necessarily improve the dispersion stability. Moreover, given that MNPs@PMPC and MNPs@PMD1 were dispersed in PBS for a shorter time than the other particles, we assumed that dispersion stability depended on DMA content. This may be attributed to the detachment of catechol groups from the polymers with fewer DMA units, which were replaced by phosphate ions in PBS. In the case of MNP@PMD5, MNP@PMD10, and MNP@PMD30, with higher DMA unit contents, it was assumed that complete detachment of the polymer did not occur and that the dispersion state was maintained because of their stable multidentate capacity.

**Figure 2:**
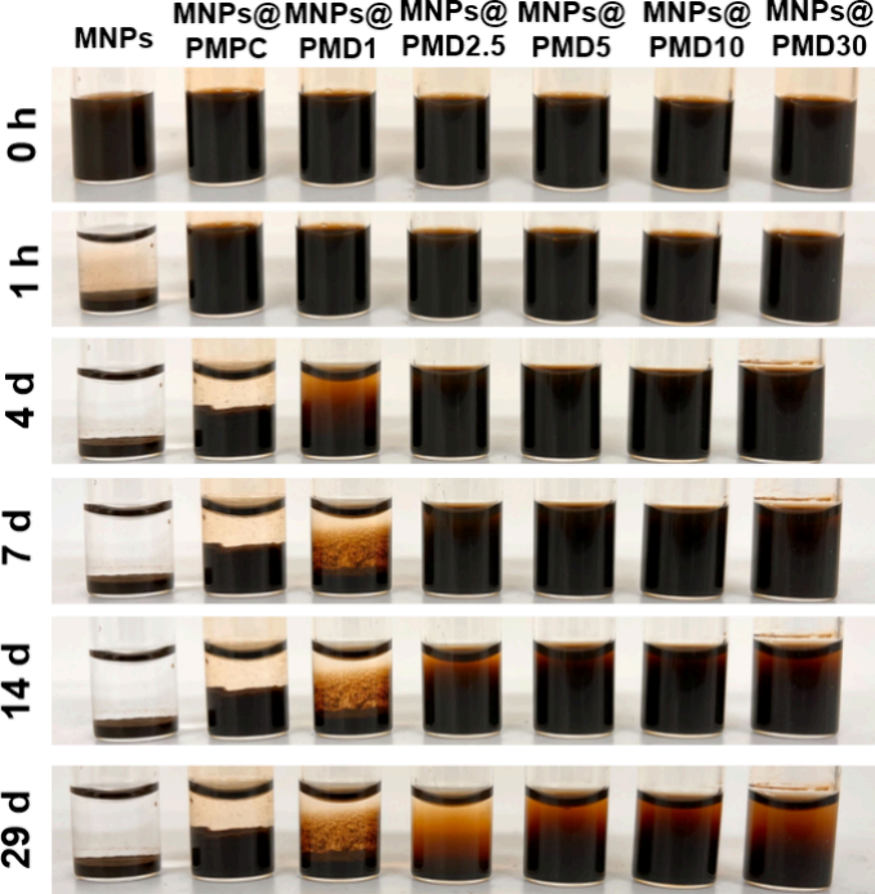
Photographs of time course changes of MNP suspensions in PBS (2.0 mg*Fe*_3_ *O*_4_/mL).

### Uptakes of MNPs by RAW264 Cells

To systemically deliver MNPs into tumor tissue, a surface structure minimally captured by a mononuclear phagocyte system, such as macrophages in the liver, must be prepared. Therefore, we investigated the effect of the PMD structure on the uptake of MNPs@PMD by mouse macrophage-like RAW264 cells. As a control, unmodified MNPs were taken up by macrophages at 18% of the total amount added to the cell culture medium after 24 h, whereas the amount of uptake was reduced by PMD modification (Figure 3). The uptake of the samples followed the order: MNPs@PMD30 > MNPs@PMD30-H > MNPs@PMD1 > MNPs@PMD1-L > MNPs@PMD2.5 > MNPs@PMD10 > MNPs@PMD5.

**Figure 3:**
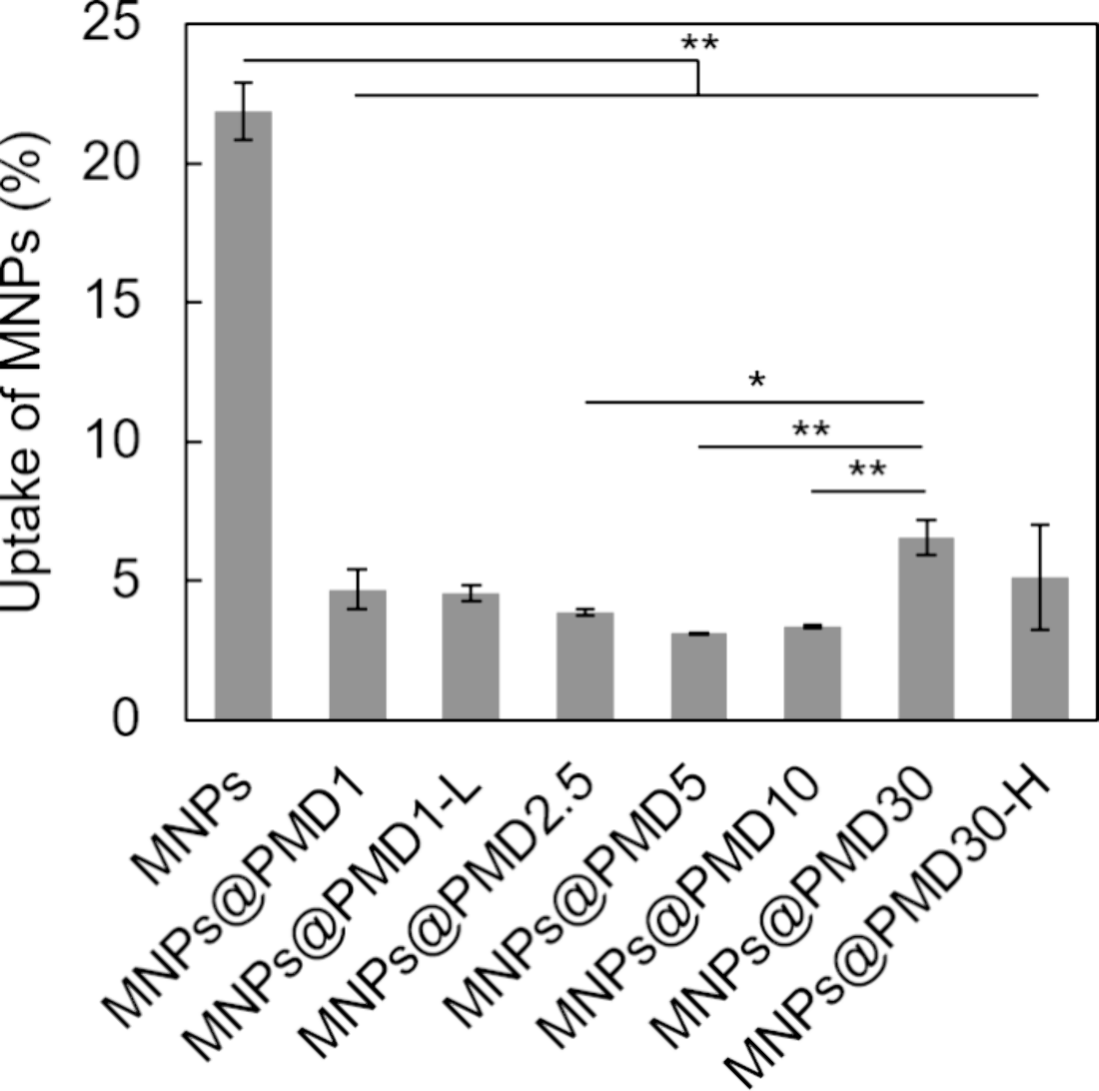
Uptakes of MNPs by RAW264 cells at 24 h after adding MNPs (0.10 mg*Fe*_3_ *O*_4_/mL). The data was expressed as mean ± SD (*n* = 3). Asterisks indicate statistically significant differences (*p < 0.05, **p < 0.01).

Figure S7 shows the effect of the ratio of DMA units in the PMD on the uptake of MNPs by macrophages, the average molecular weight of PMD, and the amount of modified polymer on the MNPs. Similar to the dispersion stability results (Figure 2), a higher modification amount did not necessarily lead to a decline in macrophage uptake (Figure S7c). In contrast, an optimal range of molecular weight and DMA unit content seemed to reduce macrophage uptake (Figure S7a,b). For MNPs@PMD with a low DMA content, the binding of the polymers to MNPs was weak because of the small number of catechol groups, which may cause detachment of the polymers, leading to increased uptake by RAW264 cells. For MNPs@PMD with a high DMA content, several catechol groups were present on the surface, which may debilitate the stealth properties of the MNPs and increase their uptake. MNPs@PMD10, which demonstrated high dispersion stability in PBS and stealth properties toward macrophages, was used for further investigation.

### In Vitro Magnetic Hyperthermia Using BTZ-Loaded Polymer-Modified MNPs

MNPs@PMD can load drugs with boronic acid groups via the catechol groups. In this study, MNPs@PMD10-BTZ were prepared by loading BTZ onto MNPs@PMD10. The hydrodynamic sizes of MNPs@PMD10 was 134 nm (Figure 4a). The larger hydrodynamic size of MNPs@PMD10 compared with that of the core MNPs (12.1 nm) suggests that the MNP aggregates were modified with PMD10. Although aggregation can affect magnetic properties, aggregated superparamagnetic iron oxide nanoparticles typically retain their superparamagnetic properties.^55,56^ While MNPs@PMD10-BTZ exhibited a similar size (129 nm) to PMD10-BTZ, they had a narrower size distribution than MNPs@PMD10, suggesting that the binding of BTZ affected the polymer conformation. The binding between the catechol and boronic acid groups can be dissociated by thermal stimulation,^26,27^ and we investigated the release characteristics of BTZ from MNPs@PMD10-BTZ after AMF irradiation (Figure 4b). The amount of BTZ released after AMF irradiation was higher than that released without irradiation, indicating that the release of BTZ was enhanced by AMF irradiation.

**Figure 4:**
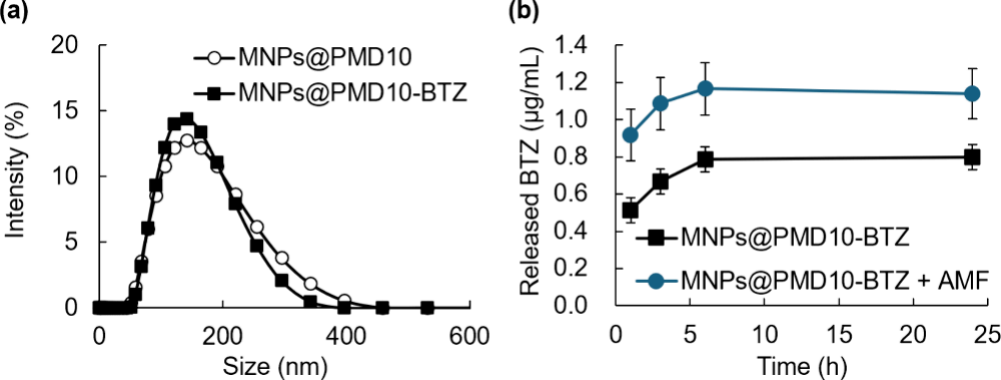
(a) Size distribution of the MNP dispersion measured by dynamic light scattering. (b) Time course of released BTZ from the MNPs@PMD10-BTZ suspensions upon AMF irradiation at 43 °C for 30 min. The data was expressed as mean ± SD (*n* = 3).

In vitro magnetic hyperthermia experiments were conducted using MNPs@PMD10-BTZ. MNPs were administered to the CT26 cell culture and irradiated with AMF for 30 min. The temperature of the medium increased to 43 °C within 5 min of AMF irradiation, which was maintained for 30 min by controlling the output of the AMF generator (Figure 5a). Figure 5b shows cell viability 24 h after AMF irradiation. Cell viability decreased with AMF irradiation; the viability of the CT26 cells treated with MNPs@PMD10-BTZ plus AMF irradiation was significantly lower than that treated with MNPs@PMD10 plus AMF irradiation. These results indicated that BTZ loading in MNPs@PMD10 enhanced its anticancer effects.

**Figure 5:**
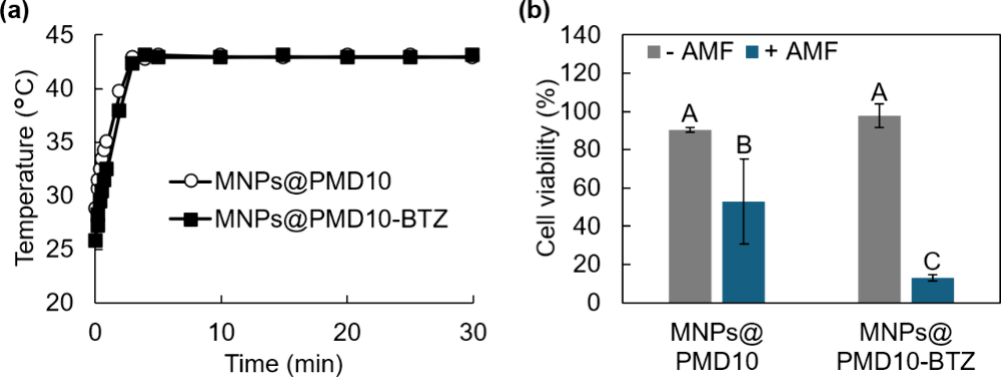
In vitro magnetic hyperthermia using MNPs@PMD-BTZ. (a) Time-course changes in temperature in the medium of CT26 cell culture during AMF irradiation. The output of the AMF generator was adjusted to maintain the temperature at 43 °C. (b) Viability of the CT26 cells after magnetic hyperthermia. The cell viability was normalized by nontreated cells. The data was expressed as mean ± SD (*n* = 3). Different letters (A, B, and C) represent statistically significant differences (*p* < 0.05), whereas groups sharing the same letter do not differ significantly.

The optimized MNPs@PMD10 demonstrated key properties essential for in vivo applications: an average particle size suitable for the EPR effect (Figure 4a), high stability in aqueous dispersions (Figure 2), and the ability to evade macrophage uptake in vitro (Figure 3). Collectively, these characteristics are expected to promote prolonged systemic circulation and enhance passive tumor accumulation via the EPR effect. However, further comprehensive evaluations are necessary for the clinical translation. While long-term dispersion stability was visually monitored in this study, future work should include more detailed analyses, such as time-dependent DLS and ζ-potential measurements. Moreover, in vivo experiments are required to assess long-term biocompatibility, pharmacokinetics, and tumor delivery efficiency. Furthermore, catechol groups on the polymer surface offer a versatile platform for advanced functionalization. In this study, they were used to load an anticancer drug, but could also be conjugated with targeting ligands, such as cancer-specific antibodies or peptides containing amine or thiol groups^57,58^ to achieve active tumor targeting and potentially improve delivery efficiency.

## Conclusions

In this study, we developed MNPs coated with catechol-containing phospholipid polymers to address cancer hyperthermia. The amount of modified polymers was positively correlated with the molecular weight of the PMD. MNPs@PMD containing more than 4.8 mol % DMA units exhibited excellent dispersion stability in PBS. Modifying the PMD reduced the uptake of MNPs by macrophages. MNPs@PMD loaded with the anticancer drug BTZ were prepared, which exhibited AMF-triggered BTZ release. In vitro magnetic hyperthermia using MNPs@PMD successfully killed CT26 cells, and BTZ loading enhanced anticancer activity. The findings of this study contribute to the development of effective cancer hyperthermia as a platform for magnetic nanoparticles with stealth properties and drug-loading capabilities.

## Supplementary Material



## Notes

^1^H NMR spectrum of PMD30; CPC chats of PMD; TEM image of MNPs; XRD pattern of MNPs; magnetization curve of MNPs; heat generation properties of MNPs; and relationship between uptakes of MNPs by macrophages and MNP properties (PDF)
