# Supplementary material for: Molecular Design of Catechol-Containing Phospholipid Polymers toward Effective Functionalization of Magnetic Nanoparticles for Cancer Hyperthermia
Source: ACS Omega. 2025 Jul 11;10(28):30708–16. doi: 10.1021/acsomega.5c02791 (PMC12290941; doi:10.1021/acsomega.5c02791)
Supplement: Supplementary file 1 [file ao5c02791_si_001.pdf]

Supporting Information

Molecular design of catechol-containing phospholipid polymers toward effective functionalization of magnetic nanoparticles for cancer hyperthermia

Masahiro Kaneko,[a*] Kanato Yukishita,[b] Kaname Tsutsumiuchi,[b] Akira Ito [a*]

* Corresponding authors

[a] *Department of Chemical Systems Engineering, Graduate School of Engineering, Nagoya University, Furo-cho, Chikusa-ku, Nagoya 464-8603, Japan*

E-mail: kaneko.masahiro@material.nagoya-u.ac.jp, ito.akira@material.nagoya-u.ac.jp

[b] *College of Bioscience and Biotechnology, Chubu University, 1200 Matsumoto, Kasugai, Aichi 487-8501, Japan*

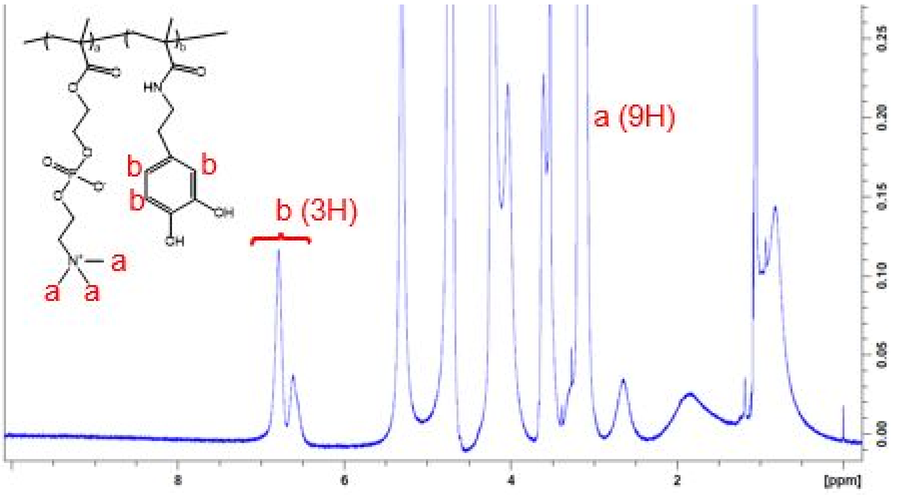

Figure S1. ^{1}HNMR spectrum of PMD30 in a mixture of D_2O/C_2D_5OD = 1/1 (v/v).

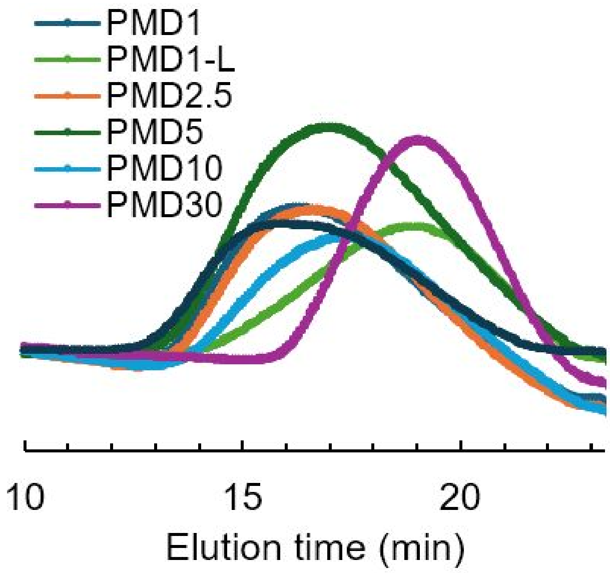

Figure S2. GPC chromatograms of PMD.

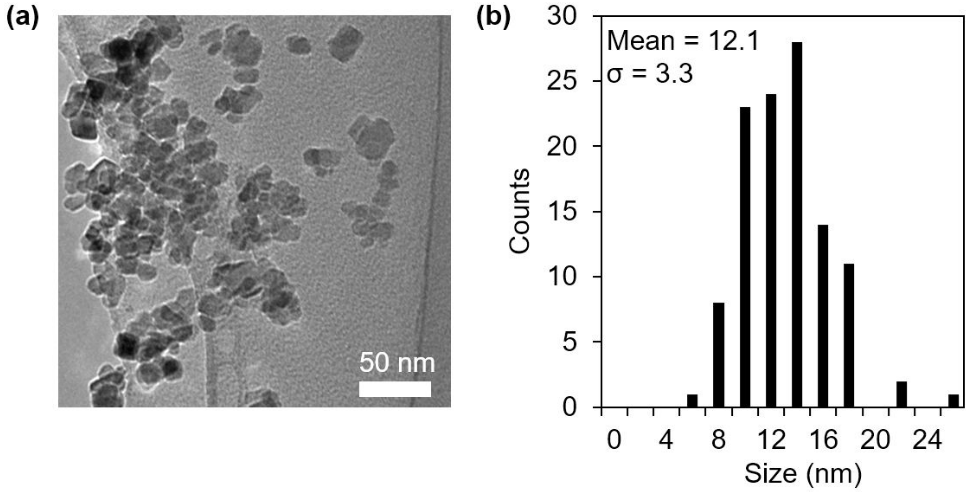

Figure S3. (a) TEM image of the bare magnetite nanoparticles (MNPs). (b) Size distribution of the MNPs determined from TEM analysis, showing a mean diameter of 12.1 ± 3.3 nm (mean ± SD; n = 112).

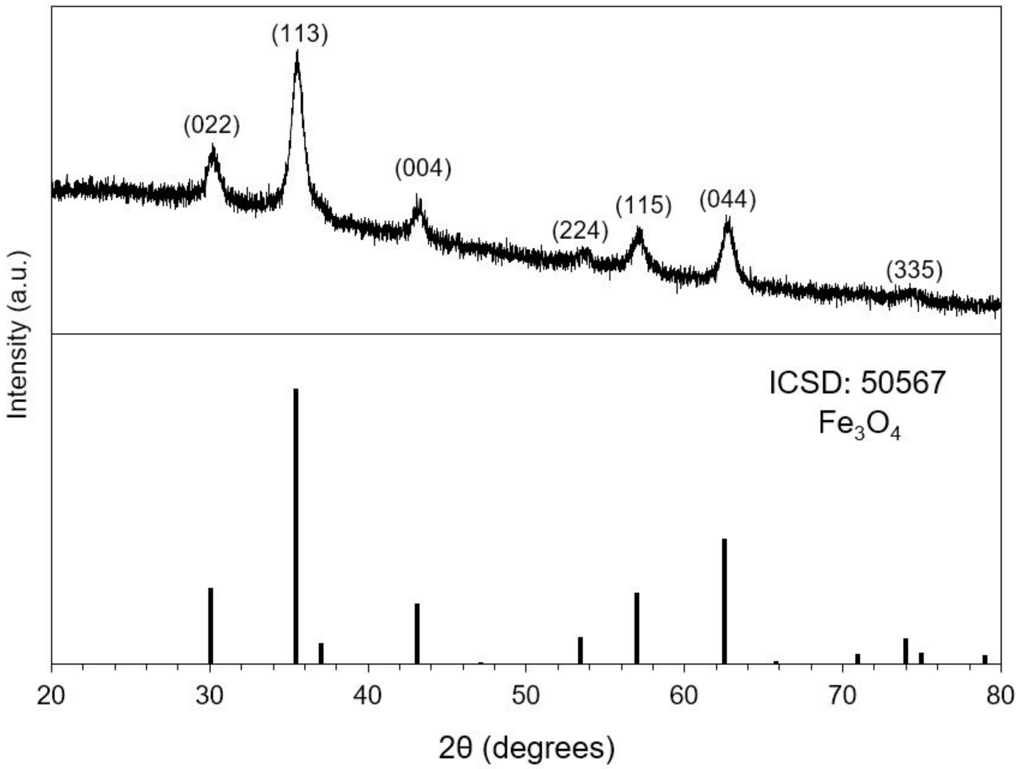

Figure S4. XRD pattern of the bare magnetite nanoparticles (MNPs) and the ICSD reference pattern (ICSD Card No. 50567).

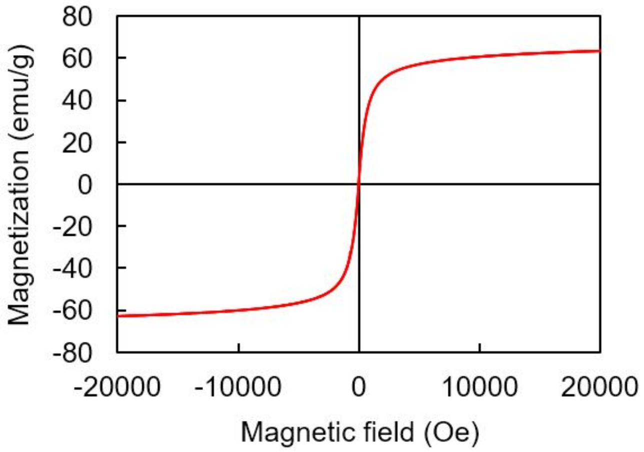

Figure S5. Magnetization curve of the bare magnetite nanoparticles (MNPs) obtained using a vibrating sample magnetometer at 300 K.

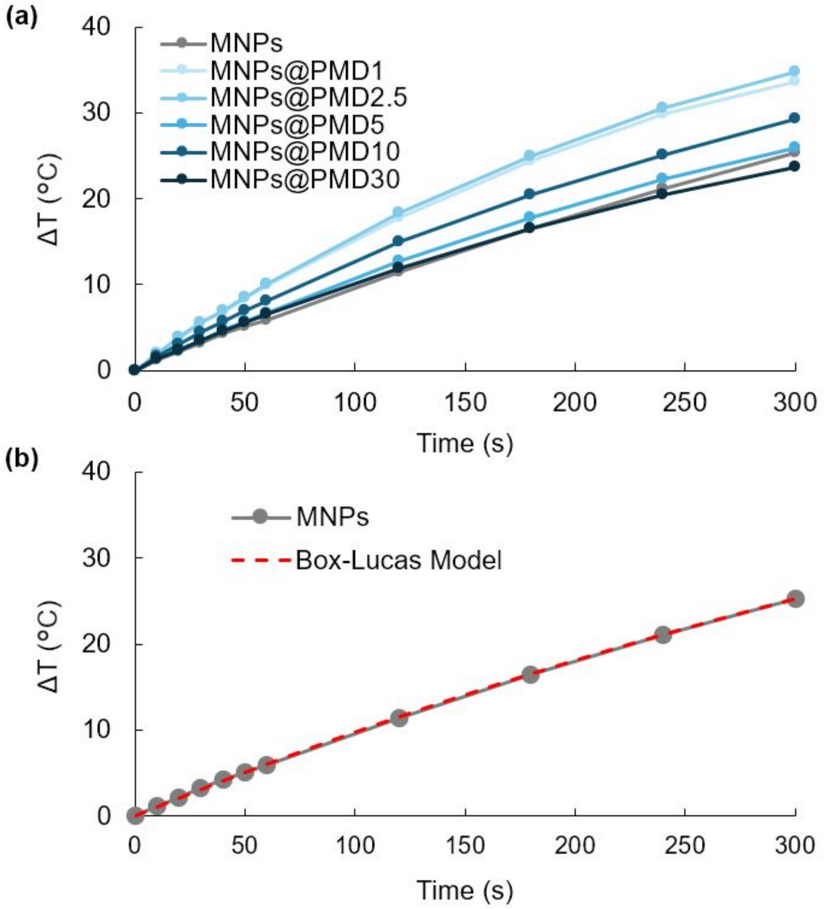

Figure S6. (a) Heat generation properties of MNPs@PMD under AMF irradiation (350 kHz, 9.5 kA/m) in pure water at 2.0 mg/mL. A fiber optic thermometer was inserted into the vial to measure the temperature of the dispersion. (b) Temperature elevation curve of the bare MNPs fitted using the Box-Lucas model.

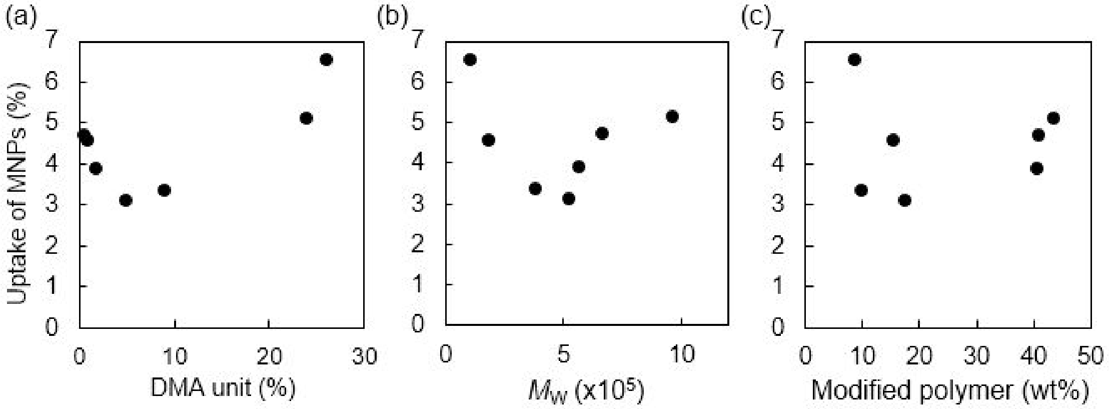

Figure S7. Effect on the uptake of MNPs by macrophages based on the (a) ratio of DMA units in PMD, (b) weight-average molecular weight (M_W) of PMD, and (c) amount of modified polymer on MNPs.
